# Peptide Modulation Overrides Glycan Synergy in Gold Nanoparticle‐Based Vaccines for Cancer Immunotherapy

**DOI:** 10.1002/cam4.71286

**Published:** 2025-10-01

**Authors:** Narumi Harada, Mayumi Niimura, Yasuhisa Sakamoto, Akihiro Nita, Mayuko Shimoda, Shiho Wada, Koki Murata, Masahiro Wakao, Tomomi Kamba, Hiroyuki Shinchi, Toshiro Moroishi

**Affiliations:** ^1^ Department of Molecular and Medical Pharmacology, Faculty of Life Sciences Kumamoto University Kumamoto Japan; ^2^ Division of Cellular Dynamics, Medical Research Laboratory Institute of Integrated Research, Institute of Science Tokyo Tokyo Japan; ^3^ Department of Engineering, Chemistry and Biotechnology Program Graduate School of Science and Engineering, Kagoshima University Kagoshima Japan; ^4^ Department of Urology Graduate School of Medical Sciences, Kumamoto University Kumamoto Japan

**Keywords:** antigen peptide, gold nanoparticle, immunotherapy, sugar chain, TLR7 ligand

## Abstract

**Background:**

We have previously developed a gold nanoparticle (GNP)‐based anti‐cancer immunotherapy, termed integrated glyco‐nanovaccine (iGN). The iGN is composed of GNPs conjugated to a synthetic toll‐like receptor (TLR) 7 ligand, an antigen peptide, and a mannose sugar chain. However, the effect of the combination of different sugar chains and antigen peptides on iGN‐mediated anticancer immunotherapy remains to be elucidated.

**Objective:**

We compared the anti‐tumor effects of two different sugar chains: α‐mannose and sialic acid.

**Results:**

We showed that not only the sugar chain but also the antigen peptide plays a pivotal role in iGN uptake by immune cells. In contrast to α‐mannose, which promoted GNP internalization by bone marrow‐derived dendritic cells (BMDC), sialic acid modification resulted in limited cellular uptake. The integration of major histocompatibility complex class I‐restricted ovalbumin peptides drastically changed this cellular recognition pattern, particularly for sialic acid‐modified iGN. The peptide largely improved the uptake of nanoparticles, delivery of the TLR 7 ligand, and subsequent activation of the type I interferon pathway in BMDC. Sialic acid‐modified iGN demonstrated comparable induction of CD8^+^ T cell and efficacy of anti‐cancer therapy to α‐mannose‐modified iGN in an EG7 syngeneic mouse tumor model.

**Conclusions:**

These results indicate that antigens, and not only the sugar chain, critically determine both the cellular internalization and immunotherapeutic efficacy of iGNs. This study presents a new design principle for glyco‐nanovaccines, where peptides override glycan synergy and determine therapeutic efficacy.

## Graphical abstract

This study explores how antigen peptides and sugar chains affect the efficacy of glyco‐nanovaccines (iGN) in cancer immunotherapy. We show that antigen peptides, rather than sugar chains, are key to nanoparticle uptake and immune activation, with sialic acid‐modified iGN showing similar anti‐cancer efficacy to α‐mannose‐modified iGN in a mouse model.

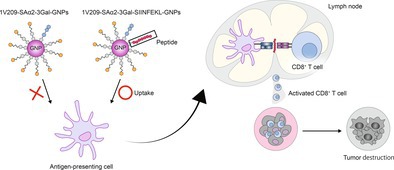

**Abbreviations**
APCs: antigen‐presenting cells
BMDC: bone marrow‐derived dendritic cells
DLS: dynamic light scattering
GNP: gold nanoparticle
ICIs: immune checkpoint inhibitors
iGN: integrated glyco‐nanovaccine
miGN: α‐mannose‐type iGN
OVA: ovalbumin
siGN: sialic acid‐type iGN
TLR: toll‐like receptor

## 1 Introduction

Cancer immunotherapy, especially with immune checkpoint inhibitors (ICIs), has shown remarkable clinical benefits in various cancers [1, 2, 3]. ICIs allow T cells to attack cancer cells more effectively [4, 5]. They can offer cancer remission, prevent recurrence by long‐term immune surveillance, and reduce side effects compared with traditional chemotherapy. However, only 20%–40% of patients who have immune active “hot tumors” can respond to ICIs, so exploring T cell activation strategies is crucial [1, 6]. Nanovaccine‐based immunotherapy aims to stimulate antigen‐presenting cells (APCs) and activate adaptive immunity to produce cancer‐specific T cells [7, 8]. Nanovaccines employ nanocarriers with diameters of less than 100 nm to efficiently deliver adjuvants and tumor antigens, thereby inducing a robust tumor‐specific immune response [9, 10]. Therefore, effective nanovaccines require the optimization of nanocarriers, adjuvants, and antigens.

Adjuvants are immunostimulants that activate the host immune response, leading to the maturation of APCs and induction of adaptive immunity against vaccines [11]. Pathogen‐associated molecular patterns, damage‐associated molecular patterns, and synthetic toll‐like receptor (TLR) ligands are widely used [12, 13]. TLRs are transmembrane proteins that play a central role in the innate immune responses triggered by pathogen‐associated molecular patterns and damage‐associated molecular patterns. Ten TLRs have been identified in humans, and 13 TLRs have been identified in mice. Various immune cells, including APCs, express TLR1, TLR2, TLR5, TLR6, and TLR10 on the plasma membrane, whereas TLR3, TLR7, TLR8, and TLR9 are on the endosomes and endoplasmic reticulum [14, 15]. TLR7 activates the type I interferon (IFN) pathway, which has anti‐cancer effects [16, 17]. Because systemic administration of TLR7 ligands causes non‐specific distribution in vivo and cytokine storms, an effective delivery system targeting APCs is required.

Nanocarriers offer targeted delivery, pharmacokinetic stabilization, enhanced cellular uptake, and reduced drug toxicity [18, 19]. Gold nanoparticles (GNPs) are promising nanocarriers owing to their high biocompatibility and ease of functionalization of the surface [20, 21]. In addition to size and shape, surface glycosylation has gained great interest because of its role in modulating functionality. Glycans (or sugar chains) are ubiquitous on the surface of organisms ranging from bacteria to human [22, 23]. These sugar chains are covalently attached to proteins (glycoproteins) and lipids (glycolipids) and play a crucial role in cellular communication. Surface glycosylation of nanomedicines has been extensively investigated, as the type and pattern of glycans can modulate the uptake, biodistribution, and pharmacokinetics [24, 25, 26]. Furthermore, glycan functionalization imparts hydrophilicity to GNPs, thereby enhancing their colloidal stability and preventing aggregation in aqueous solutions [27]. Previously, we showed that glycosylation has a great impact on the cellular uptake of GNPs [28]. Some glycans, such as α‐mannose, fucose, glucose, and N‐acetylglucosamine, promote glyco‐nanoadjuvants uptake by bone marrow‐derived dendritic cells (BMDCs), while sialic acid does not. Based on these findings, we developed an integrated glyco‐nanovaccine (iGN) combining α‐mannose, major histocompatibility complex (MHC) class I‐restricted ovalbumin (OVA) antigen peptides, and a synthetic TLR7 ligand (1V209; 2‐methoxyethoxy‐8‐oxo‐9‐(4‐carboxybenzyl)adenine) [29]. The iGN aims to enhance the target specificity and stability of nanocarriers through glycans, induce antigen‐specific adaptive immune responses with peptides, and activate the type‐I IFN pathway via the TLR7 ligand. To maximize the efficacy of iGNs, it is crucial to identify the optimal combination of glycan and antigen peptides. We investigated how various combinations of glycans and peptides within the iGN platform influence the therapeutic effectiveness against cancer.

## 2 Materials and Methods

### 2.1 Animals

Male and female C57BL/6 mice (8 weeks of age) were obtained from Kyudo Co. Ltd. (Saga, Japan) and bred in‐house. All animal experiments were conducted using mice aged 7–12 weeks. Mice were maintained under specific pathogen‐free conditions. All procedures were approved by the Institutional Animal Committee of Kumamoto University, Japan, and performed in accordance with institutional guidelines.

### 2.2 Cell Culture

Mouse T‐lymphoblast EG7 cells were kindly gifted by Dr. H. Tsukamoto from the Division of Clinical Immunology and Cancer Immunotherapy, Center for Cancer Immunotherapy and Immunobiology, Graduate School of Medicine, Kyoto University.

Cells were maintained in RPMI‐1640 medium (Wako, 189‐02025) with 10% heat‐inactivated fetal bovine serum (FBS; Nichirei, 175012), streptomycin (10 mg/mL; Wako, 168‐23191), and penicillin (10,000 U/mL) at 37°C with 5% CO_2_.

BMDCs were prepared as previously described [30]. Briefly, BMDCs from C57BL/6 mice were cultured for 9 days in RPMI‐1640 medium containing 10% heat‐inactivated FBS, streptomycin (10 mg/mL), penicillin (10,000 U/mL), and granulocyte macrophage colony‐stimulating factor (20 ng/mL; Peprotech, AF‐315‐03‐20) at 37°C with 5% CO_2_.

### 2.3 Nanoparticle Synthesis

Thioctic acid (TA)‐modified SGLEQLESIINFEKL (TA‐SGLEQLESIINFEKL) was obtained from Genscript Japan (Tokyo, Japan). TA‐modified α‐mannose (Manα1‐6Glc‐*m*PDA‐TA), TA‐modified SAα2‐3Gal (SAα2‐3Galβ1‐4Glc‐*m*PDA‐TA), and TA‐4,7,10‐trioxa‐1,13‐tridecandiamine (TTDDA)‐modified 1V209 (1V209‐TTDDA‐TA) were synthesized in the laboratory according to published protocols [28, 31, 32]. Sodium borohydride and gold (III) sodium chloride dihydrate were purchased from Nacalai Tesque (Kyoto, Japan), and Visking Tubing (MWCO: 12,000–14,000) was obtained from SERVA Electrophoresis GmbH (Germany).

1V209–αMan–GNPs and 1V209–SAα2–3Gal–GNPs were prepared by adding an aqueous solution of sodium borohydride (50 mM, 750 μL) to an aqueous solution of gold (III) sodium chloride (1.9 mM, 3975 μL), followed by vigorous stirring at room temperature for 10 min. Dimethylformamide (DMF; 2025 μL) was added to the stirred solution, followed by the addition of a 30% DMF solution of 1V209‐TTDDA‐TA and Manα1‐6Glc‐mPDA‐TA or SAα2‐3Galβ1‐4Glc‐mPDA‐TA (1.5 mM, 750 μL; molar ratio 1:9). The reaction mixture was stirred for 30 min, and excess reagents were removed by dialysis three times in water using Visking Tubing.

Peptide‐antigen‐conjugated 1V209–αMan–GNPs and 1V209–SAα2–3Gal–GNPs were prepared by adding an aqueous solution of sodium borohydride (50 mM, 750 μL) to an aqueous solution of gold (III) sodium chloride (2.2 mM, 3450 μL), followed by vigorous stirring at room temperature for 10 min. Dimethylformamide (DMF; 1800 μL) was added to the stirred solution, followed by the addition of a 30% DMF solution of TA‐SGLEQLESIINFEKL (0.15 mM, 750 μL), and subsequently, a 30% DMF solution containing 1V209‐TTDDA‐TA and Manα1‐6Glc‐mPDA‐TA or SAα2‐3Galβ1‐4Glc‐mPDA‐TA (1.35 mM, 750 μL; molar ratio 1:8) was added. After agitating the reaction mixture for 30 min, excess reagents were removed by dialysis three times in water using Visking Tubing. Hydrodynamic diameters and zeta potentials were determined using a Zetasizer Nano ZS90 (Malvern, UK).

### 2.4 Observation of GNP Uptake Into BMDCs

BMDCs (1 × 10^5^ per well) were plated in 96‐well plates, incubated to adhere for 2 h, and then stimulated with 1V209–αMan–GNPs, 1V209–SAα2–3Gal–GNPs, SGLEQLESIINFEKL–1V209–αMan–GNPs (miGN), or SGLEQLESIINFEKL–1V209–SAα2–3Gal–GNPs (siGN) for 18 h. This incubation period was based on a previous report [29] indicating retention of GNPs in lysosomes for up to 18 h. Cellular uptake was visualized using a KEYENCE BZ‐X800 microscope, and GNPs were quantified in 100 randomly selected cells with ImageJ software (NIH).

### 2.5 Immunofluorescent Staining

Glass coverslips were UV‐sterilized, placed in 12‐well plates, and coated with poly‐L‐ornithine (10 μg/mL; Wako, 163‐27421). BMDCs (1 × 10^6^ cells/well) were plated, incubated for 2 h, and stimulated with 1V209–αMan–GNPs or 1V209–SAα2–3Gal–GNPs for 2 h. This incubation period was based on a previous report indicating that NF‐κB remains in the nucleus for up to 2 h post‐stimulation [33]. Cells were fixed with 4% paraformaldehyde for 10 min, followed by permeabilization with 0.1% Triton X‐100 for 15 min. Anti‐NF‐κB p65 antibody (Cell Signaling Technology, 8242) incubation was performed overnight at 4°C. Alexa Fluor 488‐conjugated goat anti‐rabbit antibody (Invitrogen, A‐11008) was applied for 30 min at room temperature. Confocal images were acquired with an Olympus FLUOVIEW FV3000 microscope (60× oil objective lens). Nuclear‐to‐cytoplasmic NF‐κB intensity ratios were calculated from randomly 100 cells using ImageJ.

### 2.6 Reverse Transcription (RT) and Real‐Time PCR Analysis

BMDCs (1 × 10^6^ per well) were plated in 12‐well plates, cultured for 2 h, and afterwards subjected to treatment with 1V209–αMan–GNPs, 1V209–SAα2–3Gal–GNPs, SGLEQLESIINFEKL–1V209–αMan–GNPs (miGN), or SGLEQLESIINFEKL–1V209–SAα2–3Gal–GNPs (siGN) for 2 or 4 h. RNA extraction from treated BMDCs, reverse transcription, and real‐time PCR analysis were performed as previously described [29]. The qPCR primers used are as follows: (forward and reverse, respectively) 5′‐GCCTCCTTCTTGGGTATGG‐3′ and 5′‐AGGTCTTTACGGATGTCAACG‐3′ for mouse *Actb*; 5′‐CCACAGATGACATGGTGAAGACG‐3′ and 5′‐TGGTTTGGTCCCGTGTGATG‐3′ for mouse *Il12a*; 5′‐TGAAACCAGCAGCCTTTGCTC‐3′ and 5′‐AGGCATTCAGTTCCAGGTCAGTG‐3′ for mouse *Ccl3*; 5′‐GAAACAGCAGGAAGTGGGAG‐3′ and 5′‐CATGAAGCTCTGCGTGTCTG‐3′ for mouse *Ccl4*. Gene expression was normalized to that of *Actb* mRNA.

### 2.7 Flow Cytometry Analysis

To analyze CD86 expression, BMDCs (1 × 10^6^ cells/well) were plated in a 12‐well plate, incubated for 2 h, and stimulated with 1V209–αMan–GNPs, 1V209–SAα2–3Gal–GNPs, SGLEQLESIINFEKL–1V209–αMan–GNPs (miGN), or SGLEQLESIINFEKL–1V209–SAα2–3Gal–GNPs (siGN) for 12 h, followed by staining for flow cytometry analysis.

To analyze IFNγ^+^CD8^+^ cells, C57BL/6 mice were immunized by intradermal injection at the tail base with SGLEQLESIINFEKL–1V209–αMan–GNPs (miGN) or SGLEQLESIINFEKL–1V209–SAα2–3Gal–GNPs (siGN). Seven days after immunization, splenocytes isolated from the immunized mice were incubated with SIINFEKL peptide (10 μg/mL; Anaspec, AS‐60193‐1) in RPMI‐1640 medium with heat‐inactivated 10% FBS, streptomycin (10 mg/mL), and penicillin (10,000 U/mL) for 5 h at 37°C with 5% CO_2_. One hour after incubation, splenocytes were treated with a protein transport inhibitor (BD Biosciences, 555029) for 4 h, followed by staining for flow cytometry.

Cell suspensions prepared from splenocytes and BMDCs were first stained on ice for 15 min using the LIVE/DEAD Fixable Near‐IR Dead Cell Stain Kit (Invitrogen, L34976). They were then incubated with anti‐CD16/CD32 antibody (Tonbo Biosciences, 70‐0161) on ice for an additional 15 min to inhibit Fc receptor binding. Cell surface markers were stained for 30 min on ice with the following antibodies: anti‐CD8a (eFluor450, eBioscience, 48‐0081‐82), anti‐CD45 (PerCP‐Cy5.5, BD Pharmingen, 550994), anti‐CD11c (eFluor450, eBioscience, 48‐0114‐82), and anti‐CD86 (PE BioLegend, 105007). For intracellular staining, the Foxp3/Transcription Factor Staining Buffer Set (Invitrogen, 00‐5523) was used following the manufacturer's protocol. Cells were then incubated overnight at 4°C with anti‐IFNγ (APC, eBioscience, 17‐7311‐82), and analyzed by flow cytometry on a CytoFLEX S (Beckman Coulter).

### 2.8 In Vivo Cytotoxicity Assay

In vivo cytotoxicity assay was performed as described before [29]. Briefly, C57BL/6 mice were immunized by intradermal injection at the tail base with phosphate‐buffered saline (PBS), SGLEQLESIINFEKL–1V209–αMan–GNPs (miGN), or SGLEQLESIINFEKL–1V209–SAα2–3Gal–GNPs (siGN). Seven days after immunization, the in vivo cytotoxicity assay was conducted. Specific killing was calculated according to the following formula: Specific killing (%) = [1–“Sample ratio (miGN or siGN)”/“Negative control ratio in average (PBS)”] × 100; “Sample ratio (miGN or siGN)” = CFSE^low^(SIINFEKL)/CFSE^high^(Irrelevant), which means a value in each mouse immunized with miGN or siGN; “Negative control ratio in average (PBS)” = CFSE^low^(SIINFEKL)/CFSE^high^(Irrelevant), which means the average value from total mice immunized with PBS.

### 2.9 Therapeutic Experiments

EG7 cells (5 × 10^5^ cells) were subcutaneously injected into the right back flank of C57BL/6 mice (on day 0). To assess the therapeutic efficacy of miGN or siGN, mice were randomized into three groups (PBS and each iGN), and treatment was initiated 5 days post‐tumor transplantation (average tumor size, 20–30 mm^3^). Mice were injected intradermally at the tail base with PBS, SGLEQLESIINFEKL–1V209–αMan–GNPs (miGN), or SGLEQLESIINFEKL–1V209–SAα2–3Gal–GNPs (siGN) on days 5, 8, and 11. Tumors were monitored every other day to calculate the tumor volume (width^2^ × height × 0.523) using a caliper. Tumor tissues were harvested on day 15 to analyze tumor cell death.

### 2.10 Histological Analysis of iGN Target Cells

C57BL/6 mice were administered intradermally at the base of the tail with SGLEQLESIINFEKL–1V209–αMan–GNPs (miGN) or SGLEQLESIINFEKL–1V209–SAα2–3Gal–GNPs (siGN). Then, 72 h after the injection, the inguinal lymph nodes were harvested, fixed overnight at 4°C in 10% formalin neutral buffer solution (Wako, 062‐01661), and subsequently transferred to 70% ethanol for an additional overnight incubation at 4°C. Tissues were paraffin‐embedded using CT‐Pro20 (GenoStaff), followed by deparaffinization. Hematoxylin (Sakura Finetek Japan, 8656) and eosin (Sakura Finetek Japan, 8659) staining was performed according to standard procedures. Image acquisition was performed with a KEYENCE BZ‐X800 microscope (KEYENCE).

### 2.11 RNA Sequencing and Data Analysis

BMDCs (1 × 10^6^ cells per well) were plated in a 12‐well plate and incubated at 37°C with 5% CO_2_ for 2 h. BMDCs were then treated with 1V209–SAα2–3Gal–GNPs or SGLEQLESIINFEKL–1V209–SAα2–3Gal–GNPs (siGN) for 4 h, followed by total RNA extraction using RNeasy Mini Kit (Qiagen, 74104). The quality of total RNA was determined using a 4150 TapeStation System (Agilent, G2992AA). RNA was sequenced using an Illumina NovaSeq6000 sequencer (2 × 150 bp read length; San Diego, CA, USA). The quality of the raw paired‐end reads was evaluated using FastQC (version v0.12.1), and adaptor sequences were removed using Trim Galore (version 0.6.10). Paired‐end reads were mapped against mouse (GRCm39) genomes and analyzed using a series of programs, including HISAT2 (version 2.1.1), featureCounts (version 2.16.1), and DESeq2 (version 1.42.1). Expression data were further analyzed using Metascape (https://metascape.org) [33] and GSEA v4.3.3 software. HALLMARK_INTERFERON_GAMMA_RESPONSE, HALLMARK_INFLAMMATORY_RESPONSE, HALLMARK_TNFA_SIGNALING_VIA_NFKB, HALLMARK_INTERFERON_ALPHA_RESPONSE, and GOMF_CYTOKINE_ACTIVITY gene sets were obtained from the Molecular Signatures Database v4.0, distributed on the GSEA web site [34, 35]. Raw RNA‐seq data, supporting the findings of the present study, are available from the DNA Data Bank of Japan (DDBJ; accession numbers PRJDB19624).

### 2.12 Histological Analyses of Tumor

Tumor tissues were fixed with 10% neutral buffered formalin at 4°C overnight, and subsequently substituted with 70% ethanol at 4°C overnight. Paraffin embedding was performed using a CT‐Pro20 system. Tissue sections were deparaffinized and subjected to hematoxylin and eosin staining following standard protocols. Microscopic images were captured using a BZ‐X800 microscope (KEYENCE).

### 2.13 Statistical Analyses

**FIGURE 1:**
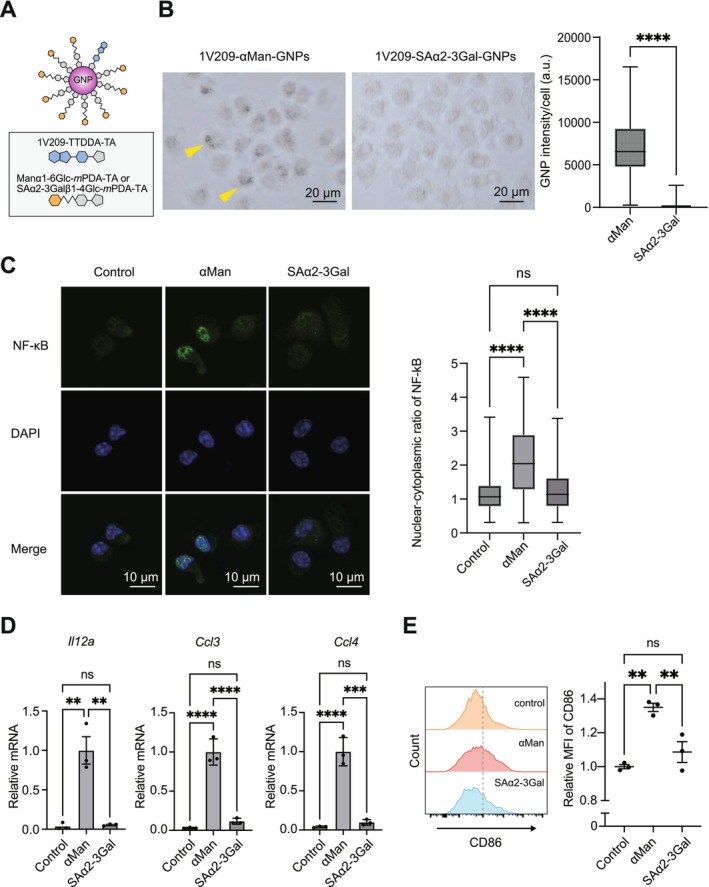
Glycan‐dependent uptake and activation of dendritic cells by nanoadjuvants. (A) Diagrammatic representation of 1V209–αMan–GNPs and 1V209–SAα2–3Gal–GNPs, with the conjugated components for each nanoparticle displayed in gray boxes. (B) GNPs uptake by the BMDCs. BMDCs were incubated with 1V209–αMan‐GNPs or 1V209–SAα2–3Gal–GNPs for 18 h. The cells were observed using a bright‐field microscopy. Right panel: GNP signals for individual cells in box and whisker plot (median, 25th and 75th percentiles, and whiskers extending to minimum and maximum values). For the quantification of GNP uptake, 100 cells were randomly selected. ****p < 0.0001, unpaired two‐tailed *t*‐test. (C) Nuclear translocation of NF‐κB induced by GNPs with different sugar chains. BMDCs were stimulated with 1V209–αMan–GNPs or 1V209–SAα2–3Gal–GNPs for 2 h. NF‐κB subcellular localization was evaluated using immunofluorescence staining for NF‐κB (green) with DAPI for nucleus (blue). Representative images are shown in the left panel. Right panel: Nuclear‐to‐cytoplasmic ratio of NF‐κB signal intensity for individual cells in box and whisker plot (median, 25th and 75th percentiles, and whiskers extending to minimum and maximum values). For the quantification of NF‐κB localization analysis, 100 cells were randomly selected. ****p < 0.0001, One‐way ANOVA with Tukey's multiple comparison test. Scale bar: 10 μm. (D) Comparison of NF‐κB target gene expression induced by GNPs with different sugar chains. BMDCs were stimulated with 1V209–αMan–GNPs or 1V209–SAα2–3Gal–GNPs for 4 h, followed by reverse transcription (RT) and real‐time PCR analysis for *Il12a*, *Ccl3*, and *Ccl4*. Expression of mRNA was adjusted to *Actb* and presented relative to 1V209–αMan–GNPs‐stimulated BMDCs. *n* = 3 per group; biological replicates. Data are presented as means ± SEM; **p < 0.01, ***p < 0.001, ****p < 0.0001, One‐way ANOVA with Tukey's multiple comparison test. (E) BMDC activation markers stimulated by GNPs with different sugar chains. BMDCs were treated with 1V209–αMan–GNP or 1V209–SAα2–3Gal–GNP for 12 h, and CD86 expression was then evaluated using flow cytometry. Relative mean fluorescence intensity (MFI) of CD86 is indicated. *n* = 3 for each group and biological replicates. Data are presented as means ± SEM; **p < 0.01, One‐way ANOVA with Tukey's multiple comparison test. BMDC, bone marrow‐derived dendritic cells; GNP, gold nanoparticle.

**FIGURE 2:**
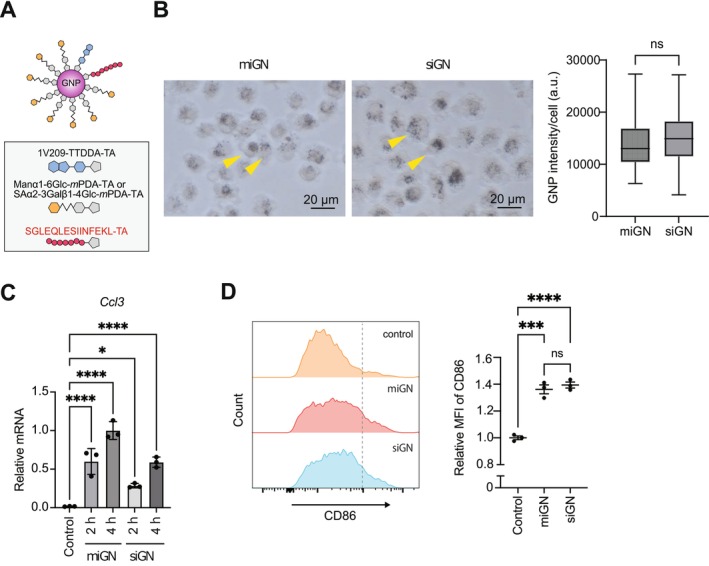
Antigen peptide integration equalizes the uptake efficacy of different glyco‐nanoadjuvants. (A) Schematic diagram of miGN and siGN. The conjugated components for each nanoparticle displayed in gray boxes. (B) iGNs uptake into BMDCs. BMDCs were incubated with miGN or siGN for 18 h. The cells were observed using bright field microscopy. Right panel: GNP signals for individual cells in box and whisker plot (median, 25th and 75th percentiles, and whiskers extending to minimum and maximum values). For the quantification of GNP uptake, 100 cells were randomly selected. ns, not significant, unpaired two‐tailed *t*‐test. (C) Expression of NF‐kB target gene *Ccl3* induced by miGN or siGN. BMDCs were stimulated with miGN or siGN for 2 or 4 h, followed by reverse transcription (RT) and quantitative PCR analysis of *Ccl3* expression. Gene expression was normalized to *Actb* and displayed relative to miGNP‐stimulated BMDCs at 4 h. *n* = 3 for each group, biological replicates. Data are presented as means ± SEM; *p < 0.05, ****p < 0.0001. One‐way ANOVA test with Dunnett's multiple comparisons test. (D) Expression of the co‐stimulatory molecule CD86 induced by miGN or siGN. BMDCs were treated with miGN or siGN for 12 h for the analysis of CD86 expression by flow cytometry. Relative mean fluorescence intensity (MFI) of CD86 was quantified. *n* = 3 for each group, biological replicates. Data are presented as means ± SEM; ***p < 0.0001, ****p < 0.0001, One‐way ANOVA test with Tukey's multiple comparison test. BMDC, bone marrow‐derived dendritic cells; GNP, gold nanoparticle.

Statistical analyses were conducted using GraphPad Prism version 9 (GraphPad Software, La Jolla, CA, USA). Details of the statistical methods and parameters are provided in the corresponding Figures and Figure legends. Differences between two groups were assessed using an unpaired two‐tailed *t*‐test (Figures 1B and 2B). For comparisons among three groups, one‐way or two‐way ANOVA followed by Tukey's multiple comparisons test was applied (Figures 1D,E, 2D, 4D,F and 5B,C). To compare each treatment group with the control, a one‐way ANOVA followed by Dunnett's multiple comparisons test was used (Figure 2C). *p* value < 0.05 was considered statistically significant.

## 3 Results

### 3.1 Glycan‐Dependent Uptake and Activation of Dendritic Cells by Nanoadjuvants

In our previous studies, we synthesized glyco‐nanoadjuvants consisting of glycan and the TLR7 ligand 1V209, specifically 1V209–αMan–GNPs and 1V209–SAα2–3Gal–GNPs [28]. The synthetic TLR7 ligand 1V209 was immobilized on GNPs using TTDDA and TA (Figure S1A). Additionally, α‐mannose and sialic acid‐α2‐3‐galactose were immobilized on GNPs through glucose, *m*PDA, and TA, referred to as Manα1‐6Glc‐*m*PDA‐TA and SAα2‐3Galβ1‐4Glc‐*m*PDA‐TA, respectively (Figure 1A and Figure S1B–D). The proportion of each component on GNPs was as follows: 1V209: sugar moieties = 1: 9 (Figure S1E). Transmission electron microscopy and dynamic light scattering (DLS) analyses revealed that the size of 1V209–SAα2–3Gal–GNPs was slightly larger than that of 1V209–αMan–GNPs, with an average diameter of 9.4 and 8.4 nm in DLS, respectively (Figure S1F,G). To investigate the impact of different glycans on GNP uptake by immune cells, we conducted an in vitro GNP uptake assay using BMDCs. We found that 1V209–αMan–GNPs and 1V209–SAα2–3Gal–GNPs are differently recognized by BMDCs. α‐Mannose led to the uptake of GNPs, but sialic acid did not (Figure 1B), which is consistent with our previous observations [28]. Although we showed different internalization of 1V209–αMan–GNPs and 1V209–SAα2–3Gal–GNPs, the activation of intracellular signaling post‐uptake remained unclear. To evaluate the consequences of differential GNP uptake, we investigated the immune responses of BMDCs. The TLR7 ligand 1V209 activates the TLR7–MYD88 pathway [36, 37], followed by the nuclear translocation of nuclear factor kappa B (NF‐κB) [38, 39]. We found that treatment with 1V209–αMan–GNPs induced NF‐κB nuclear translocation compared with the control. In contrast, 1V209–SAα2–3Gal–GNPs did not enhance NF‐κB nuclear translocation (Figure 1C). Furthermore, pro‐inflammatory cytokine genes targeted by NF‐κB, such as *Il12a* [40], *Ccl3* [41, 42], and *Ccl4* [42] were markedly upregulated by stimulation with 1V209–αMan–GNPs, but not with 1V209–SAα2–3Gal–GNPs or the control (PBS treatment) (Figure 1D). These results indicate that 1V209–αMan–GNPs are taken up by BMDCs and activate the NF‐κB pathway, whereas 1V209–SAα2–3Gal–GNPs do not. To determine whether these glyco‐nanoadjuvants induced BMDC maturation, we examined the expression of the co‐stimulatory factor CD86, which is crucial for antigen presentation to T cells [42]. Flow cytometry analysis showed that CD86 expression was increased in BMDCs treated with 1V209–αMan–GNPs but not with 1V209–SAα2–3Gal–GNPs. Taken together, conjugation of α‐mannose, compared with sialic acid, enhances GNP uptake, thereby stimulating TLR7 and triggering an inflammatory response. These results indicate that glycans play a critical role in determining cellular uptake and subsequent immunological activation by GNPs.

### 3.2 Antigen Peptide Integration Equalizes the Uptake Efficacy of Different Glyco‐Nanoadjuvants

While specific glycans conjugation confers cellular uptake of GNPs and subsequent immune activation, the contribution of antigen peptides to the cellular uptake and immunological behavior of glyco‐nanovaccines remains to be elucidated. To investigate the impact of peptide conjugation to glyco‐nanoadjuvant, we developed iGNs conjugating the model antigen OVA “SIINFEKL,” which binds to mouse H‐2Kb MHC class I molecules, to α‐mannose‐ or sialic acid‐conjugated glyco‐nanoadjuvants. Following the established synthetic protocol for α‐mannose‐type iGN (referred to as miGN), the long OVA peptide sequence “SGLEQLESIINFEKL” was conjugated to 1V209–SAα2–3Gal–GNP to produce sialic acid‐type iGN (referred to as siGN) (Figure 2A and Figure S2A,B). The proportion of each component on GNPs was as follows: the OVA peptides: 1V209: sugar moieties = 1: 1: 8 (Figure S2C). Transmission electron microscopy and DLS analysis confirmed that the sizes of miGN and siGN were similar, with an average diameter of 11.9 and 12.3 nm, respectively, as measured using DLS (Figure S2D,E). We also analyzed the zeta potential of these particles to assess the impact of peptide conjugation on their surface charge. While 1V209–SAα2–3Gal–GNPs exhibited a highly negative zeta potential (approximately −30 mV), conjugation with the peptide “SGLEQLESIINFEKL” reduced the surface charge of siGN to a level similar to that of miGN (approximately −20 mV), indicating that the peptide modulates the nanoparticle surface properties (Figure S3).

Next, we conducted an in vitro uptake assay using both the iGN variants. In contrast to the observations with each glyco‐nanoadjuvant (Figure 1B), we found that both miGN and siGN were similarly internalized by BMDCs (Figure 2B). These results indicated that the addition of the peptide markedly enhanced siGN uptake compared with the uptake without the peptide. We then examined whether iGNs stimulated the production of proinflammatory cytokines. *Ccl3* expression increased in a time‐dependent manner for both types of iGNs, indicating that siGNs activate the TLR7 signaling pathway (Figure 2C). Furthermore, CD86 expression increased comparably in both the miGN and siGN groups (Figure 2D). These results suggest that the integration of the model antigen peptide with glycans in glyco‐nanoadjuvants overrides the effects of glycans on cellular incorporation and activation.

### 3.3 Gene Expression Profiling Reveals Immune Activation in BMDCs Stimulated by siGN

**FIGURE 3:**
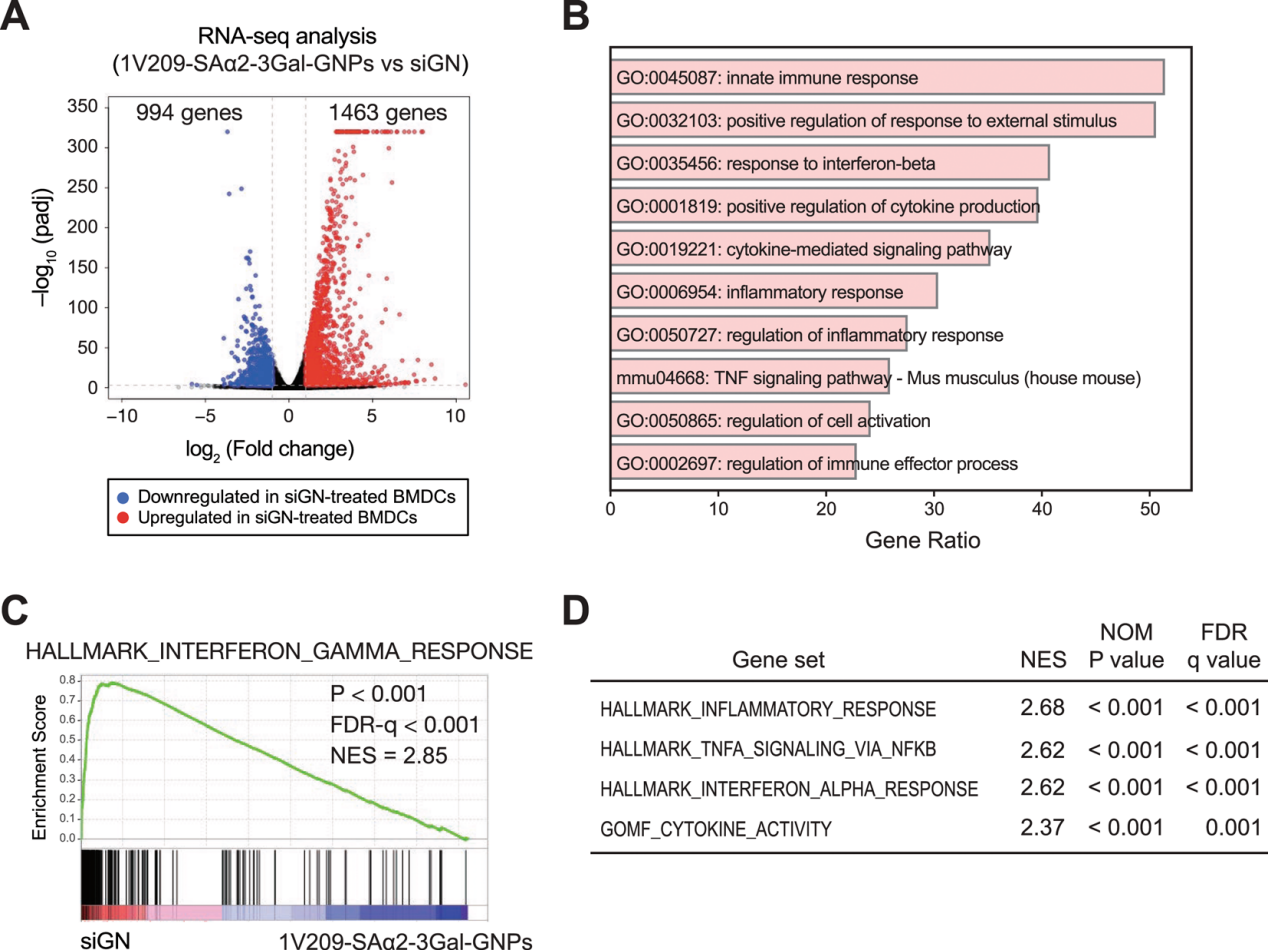
Gene expression profiling reveals immune activation in BMDCs stimulated by siGN. (A) Volcano plot of differentially expressed genes (DEGs; log2[fold change] of > 1 or < −1, and adjusted *p* < 0.001) in the primary BMDCs treated with siGN comparing with 1V209–SAα2–3Gal–GNP (control). The genes with downregulated and upregulated expression are indicated in blue and red, respectively. (B) Gene ontology analysis of the 1463 upregulated genes of 1V209–SAα2–3Gal–GNP‐ (control) and siGN‐treated BMDCs. (C) Gene set enrichment analysis (GSEA) of the HALLMARK_INTERFERON_GAMMA_RESPONSE in the primary BMDCs treated with siGN comparing with 1V209–SAα2–3Gal–GNP (control). FDR‐q, false discovery rate q value; NES, normalized enrichment score. (D) Summary of GSEA results for gene sets related to the inflammatory response or BMDC activation. BMDC, bone marrow‐derived dendritic cells; GNP, gold nanoparticle; NOM, nominal.

To investigate the impact of peptide conjugation to glyco‐nanoadjuvant on BMDC activation, we examined the gene expression profiles in siGN‐treated BMDCs compared with 1V209–SAα2–3Gal–GNP‐treated (control) BMDCs (Tables S1 and S2). Volcano plot analysis identified 2457 differentially expressed genes: 994 downregulated and 1463 upregulated genes with a log2[fold change] of > −1 or < 1 and an adjusted *p* value < 0.001 in cells stimulated with siGN compared with control cells (Figure 3A, Table S3). We then performed Gene Ontology analysis on 1463 upregulated genes and evaluated the gene expression profile using Gene Set Enrichment Analysis. GO analysis suggested an increase in innate immune response and cytokine production in BMDCs (Figure 3B). Gene Set Enrichment Analysis with an IFNα response gene set revealed a significant enrichment of immune activation in BMDCs treated with siGN (Figure 3C). Additionally, a similar activation of immune responses was observed for the other gene sets (Figure 3D). These data indicated that integrating the model antigen peptide “SIINFEKL” into 1V209–SAα2–3Gal–GNP promotes cellular uptake of siGN, triggering inflammatory‐ and interferon‐related immune responses.

### 3.4 Antigen‐Conjugated α‐Mannose‐ and Sialic Acid‐Type Glyco‐Nanovaccines Induce Comparable Immune Responses In Vivo

**FIGURE 4:**
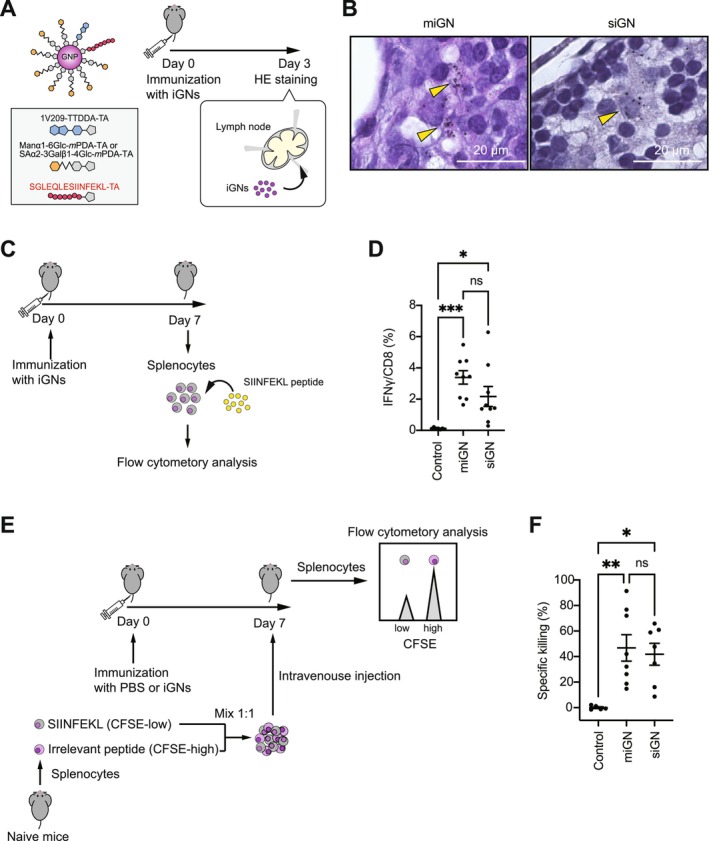
Antigen‐conjugated α‐mannose‐ and sialic acid‐type glyco‐nanovaccines induce comparable immune responses in vivo. (A) Schematic representation of iGNs uptake assay into lymph nodes is shown. Inguinal lymph nodes were harvested 72 h following immunization with each iGNs (0.4 nmol/injection, based on 1V209 amount). (B) Inguinal lymph nodes from C57BL/6 mice injected with each iGN were subjected to hematoxylin and eosin (H&E) staining. The arrowheads indicate each iGN accumulation. Scale bars, 20 μm. (C) Schematic representation of T cell activation assay. IFNγ expression in CD8^+^ T cells isolated from splenocytes of mice immunized with PBS or each iGNs (0.4 nmol/injection, based on 1V209 amount) was evaluated. At 7 days post‐inoculation, splenocytes were pulsed with SIINFEKL peptide and analyzed using flow cytometry. (D) The frequency of IFNγ^+^ cells induced by SIINFEKL peptide stimulation in the CD8^+^ T cell population was quantified 7 days post immunization. *n* = 7–9 mice for each group. Data are presented as means ± SEM; *p < 0.05, ***p < 0.001, One‐way ANOVA test with Tukey's multiple comparison test. (E) Schematic illustration of the in vivo cytotoxicity assay based on carboxyfluorescein succinimidyl ester (CFSE) labeling. SIINFEKL peptide‐ or irrelevant peptide‐pulsed splenocytes were labeled with each CFSE concentration, mixed at a 1:1 ratio, and injected into mice immunized with PBS or iGNs (0.4 nmol/injection, calculated as 1V209 content). Five hours later, splenocytes from the mixed cell‐injected mice were analyzed by flow cytometry to determine the ratio of CFSE‐low and CFSE‐high populations. (F) Antigen‐specific killing activity was calculated. *n* = 5–8 mice for each group. Data are presented as means ± SEM; *p < 0.05, **p < 0.01, One‐way ANOVA test with Tukey's multiple comparison test.

We have previously shown that miGN is taken up by APCs in tumor‐draining inguinal lymph nodes to stimulate an immune response [29]. Therefore, we investigated whether siGN exhibits a similar distribution in vivo. miGN and siGN were intradermally injected into the tail bases of C57BL/6 mice, and the inguinal lymph nodes were collected 3 days later (Figure 4A). Histological analysis revealed that both miGN and siGN accumulated in large cells, presumably in APCs. These results suggest that both miGN and siGN are taken up by APCs and stimulated in the inguinal lymph nodes. Activated APCs prime antigen‐specific CD8^+^ T cells in draining lymph nodes [4]. As we previously reported that miGN induces antigen‐specific CD8^+^ T cells [29], we examined whether siGN could also induce antigen‐specific CD8^+^ T cells in vivo. C57BL/6 mice were immunized with miGN or siGN, and 1 week later, splenocytes were stimulated with the SIINFEKL peptide to evaluate antigen‐specific IFNγ production in CD8^+^ T cells (Figure 4C). Both miGN‐ and siGN‐injected mice exhibited a comparable increase in IFNγ^+^CD8^+^ T cells compared with the control (PBS‐treated) mice (Figure 4D), indicating that miGN and siGN can induce T cell activation. To further evaluate antigen‐specific cytotoxic T cell activity induced by iGN treatment, we performed an in vivo cytotoxicity assay (Figure 4E). Immunization with either miGN or siGN resulted in an increase in cytotoxic activity comparable to that of the control (Figure 4F). Taken together, our data indicate that intradermal administration of either siGN or miGN stimulates antigen‐specific cytotoxic responses in vivo.

### 3.5 Antigen‐Conjugated Glyco‐Nanovaccines Induce Effective Anti‐Cancer Immune Responses

**FIGURE 5:**
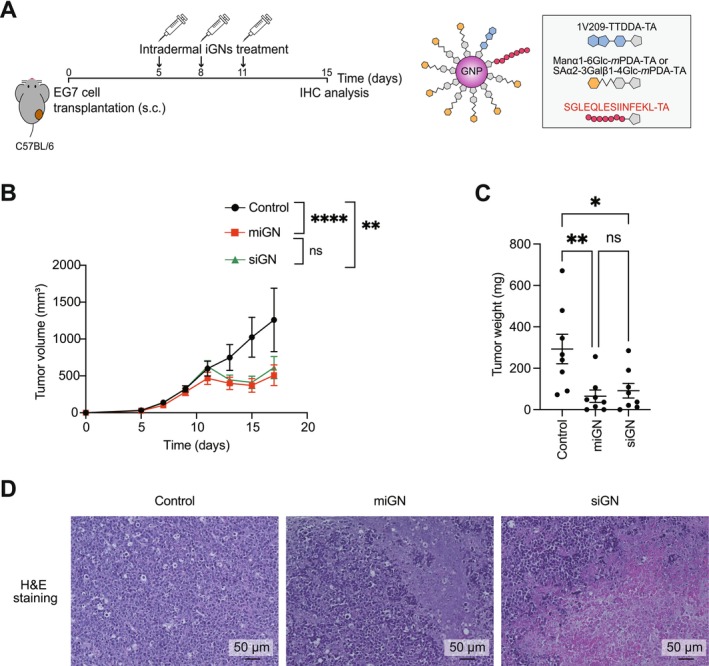
Antigen‐conjugated glyco‐nanovaccines induce effective anti‐cancer immune responses. (A) Experimental design for assessing anti‐cancer effects of miGN or siGN in a syngeneic cancer model. C57BL/6 mice were subcutaneously inoculated with 5 × 10^5^ EG7 cells in the right flank. Beginning on day 5 post‐inoculation, mice received intradermal injections of PBS, miGN, or siGN (0.4 nmol per dose, based on 1V209 content) at the base of the tail every 3 days for a total of three administrations (on days 5, 8, and 11). (B) Tumor progression in each group was measured from day 5 to day 17. *n* = 8–9 mice for each group. Data are presented as means ± SEM; **p < 0.01, ****p < 0.0001, two‐way ANOVA test with Tukey's multiple comparison test. (C) Tumor weight in each group was measured on day 15. *n* = 8 mice for each group. Data are presented as means ± SEM; *p < 0.05, **p < 0.01, one‐way ANOVA test with Tukey's multiple comparison test. (D) Hematoxylin and eosin (H&E) staining was performed on paraffin‐embedded tumors collected 15 days post‐transplantation. Scale bars, 50 μm.

Given that vaccination with siGN or miGN induces an antigen‐specific cytotoxic response, we hypothesized that these glyco‐nanovaccines might induce host immune responses against cancer. To test this hypothesis, we examined the therapeutic effects of iGN in a murine syngeneic tumor model of EG7, a mouse T lymphoma cell line expressing OVA. EG7 cells were transplanted into the back flanks of mice, followed by intradermal administration of iGN at the tail base three times every 3 days (on days 5, 8, and 11 after transplantation) (Figure 5A). In both the miGN‐ and siGN‐treated groups, tumor growth retardation became apparent on day 11, with noticeable tumor reduction effects observed until day 17 compared with the PBS‐treated control group (Figure 5B). On day 15, comparable tumor mass reduction (Figure 5C) and massive tumor destruction (Figure 5D) were observed in both the miGN and siGN groups compared with the PBS control group. These results indicate that siGN achieved therapeutic effects equivalent to those of miGN, suggesting that antigen peptide conjugation to glyco‐nanoadjuvants overrides the effects of glycan on immune induction and the therapeutic efficacy of glyco‐nanovaccines against cancer.

## 4 Discussion

Development of effective drug delivery systems for cancer vaccines is crucial for advancing cancer immunotherapy [43]. In this study, we evaluated the effectiveness of iGNs in modulating immune responses, focusing particularly on the combination of antigen peptides with various sugar chains, such as α‐mannose and sialic acid glycans. Our results demonstrate that integrating the OVA peptide into glyco‐nanovaccines results in comparable efficacy for both miGN and siGN, irrespective of the glycan type used. Both miGN and siGN effectively activate TLR7 signaling and elicit similar inflammatory responses in BMDCs. Additionally, they accumulate in APCs within the lymph nodes, stimulate antigen‐specific CD8^+^ T cell responses, induce cytotoxic T cell activity, and suppress tumor growth in murine models. These findings suggest that, although the initial glycan choice of glyco‐nanoadjuvants affects GNP uptake by APCs, the inclusion of antigen peptides can neutralize glycan‐induced differences, leading to similar immunological outcomes.

In the earlier part of our study, we showed that α‐mannose‐modified GNPs (1V209–αMan–GNPs) exhibited enhanced uptake compared with their sialic acid‐modified counterparts (1V209–SAα2–3Gal–GNPs) (Figure 1B). These results are consistent with our previous studies showing that α‐mannose‐modified GNPs are preferentially recognized and internalized by dendritic cells through the mannose receptor (MR/CD206), as excess mannans compete with the cellular uptake of α‐mannose‐modified nanoparticles [28]. In contrast, sialic acid‐modified GNPs tend to evade phagocytic uptake by interacting with sialic acid‐binding immunoglobulin‐like lectins [28, 44, 45]. The contrasting effects of mannose and sialic acid on cellular uptake were attributed to their distinct roles in immune recognition. Mannose, often found on the surfaces of pathogens, is recognized as a “non‐self” marker by immune cells, leading to its internalization by dendritic cells. In contrast, sialic acid is a common component of vertebrate cell surfaces, acting as a “self” marker and generally reducing immune cell uptake and activation [46, 47]. Integration of the MHC class I‐restricted OVA peptide antigen effectively neutralized these differences in uptake, resulting in similar internalization for both miGN and siGN (Figure 2B). These results suggest that antigen‐peptide integration can override glycan‐dependent uptake mechanisms.

The current study does not address the underlying molecular mechanisms behind peptide‐enhanced GNP uptake. It is possible that the peptide interacts with other cellular receptors or alters the physical characteristics of the GNPs. Burgdorf et al. demonstrated that mannose receptors mediate the internalization of OVA antigen by APCs [48, 49]. To directly test this hypothesis, future studies could compare the uptake and immunostimulatory capacity of OVA‐peptide‐conjugated siGNs in mannose receptor–deficient dendritic cells.

To evaluate the impact of peptide conjugation on the physical properties of the GNPs, we measured their zeta potential (Figure S3). The 1V209–αMan–GNPs exhibited an almost neutral surface charge (approximately −5 mV), consistent with the uncharged nature of mannose. In contrast, 1V209–SAα2–3Gal–GNPs displayed a strong negative charge (approximately −30 mV), likely due to the negatively charged sialic acid. Conjugation of the OVA peptide to these GNPs significantly altered their surface charge. Both miGN and siGN exhibited a similar, modest negative charge (approximately −20 mV). Given that the peptide molecule is larger than the glycan moieties, it is likely to extend from the nanoparticle surface, thereby contributing to the overall zeta potential of the GNPs. In siGN, the peptide may counteract the strong negative charge of sialic acid, resulting in a zeta potential that is less negative than that of 1V209–SAα2–3Gal–GNPs. Given the negatively charged nature of cell surfaces, we speculate that nanoparticles with a reduced negative surface potential may be more readily taken up. This idea is supported by previous studies demonstrating that positively charged nanoparticles interact more efficiently with negatively charged cell membranes, thereby enhancing cellular uptake [50]. In addition to surface charge, peptide hydrophobicity also influences nanoparticle behavior. For instance, peptide hydrophobicity could induce nanoparticle aggregation via interparticle interactions, which may enhance cellular uptake. However, DLS analysis revealed that both miGN and siGN exhibited monodisperse size distributions in PBS, suggesting that such interactions are minimal in the case of OVA peptides. In conclusion, peptide conjugation could facilitate cellular uptake of GNPs by neutralizing their surface charge. However, the zeta potential could also be influenced by the peptide's isoelectric point, and it remains unknown whether this peptide‐override effect can be generalized to other antigen peptides. Future studies should focus on optimizing peptide length and amino acid composition to improve the surface charge of nanoparticles and enhance vaccine performance.

GNPs have been applied in clinical trials owing to their high biocompatibility and accessibility to surface functionalization [20, 21]. In several studies, peptide‐modified GNPs have demonstrated favorable safety profiles. For instance, human proinsulin peptide‐conjugated GNPs achieved therapeutic efficacy with minimal hypersensitivity reactions following intradermal administration via microneedles [51]. Additionally, GNP‐based vaccines conjugated with dengue or SARS‐CoV‐2 peptides have entered clinical evaluation [52, 53]. These findings suggest that GNPs are compatible not only with model antigens such as OVA, but also with clinically relevant pathogen‐derived peptides such as patient‐specific tumor antigens.

The OVA/EG7 model employed in this study consists of EL4 murine T lymphoma cells engineered to express OVA, and is widely used to study MHC class I‐mediated antigen presentation and CD8^+^ T cell responses. Although OVA is not a tumor antigen in humans, and this model does not recapitulate the complexity of the human tumor microenvironment or antigen heterogeneity, it serves as a robust system for proof‐of‐concept evaluation in preclinical study. To take advantage of this strategy toward clinical application, combination therapies with immune checkpoint inhibitors (ICIs) should be explored in future study. For broader validation, additional tumor models—particularly immunologically “cold” tumors such as B16 melanoma—should also be considered.

In conclusion, our findings suggest that both α‐mannose and sialic acid‐based glyco‐nanovaccines could serve as viable platforms for cancer immunotherapy when properly designed with integrated antigens. Flexibility in glycan selection offers the potential to optimize other aspects of vaccine design, including stability, production costs, and tissue‐specific targeting. This study paves the way for the rational design of vaccines that harness the interplay between glycans and peptides, thereby offering new opportunities to improve cancer immunotherapy.

## Author Contributions

**Narumi Harada:** conceptualization (equal), data curation (equal), formal analysis (equal), investigation (equal), methodology (equal), validation (equal), visualization (equal), writing – original draft (equal), writing – review and editing (equal). **Mayumi Niimura:** conceptualization (equal), data curation (equal), formal analysis (equal), investigation (equal), methodology (equal), validation (equal), visualization (equal), writing – original draft (equal), writing – review and editing (equal). **Yasuhisa Sakamoto:** supervision (supporting), visualization (supporting), writing – original draft (equal), writing – review and editing (equal). **Akihiro Nita:** formal analysis (equal), visualization (equal), writing – review and editing (equal). **Mayuko Shimoda:** data curation (supporting), investigation (supporting), writing – review and editing (equal). **Shiho Wada:** data curation (equal), resources (equal), writing – review and editing (equal). **Koki Murata:** data curation (equal), resources (equal), writing – review and editing (equal). **Masahiro Wakao:** data curation (supporting), methodology (equal), resources (supporting), writing – review and editing (equal). **Tomomi Kamba:** supervision (supporting), writing – review and editing (equal). **Hiroyuki Shinchi:** conceptualization (equal), data curation (equal), formal analysis (equal), investigation (equal), methodology (equal), resources (equal), supervision (equal), validation (equal), visualization (equal), writing – review and editing (equal). **Toshiro Moroishi:** conceptualization (equal), formal analysis (supporting), funding acquisition (equal), methodology (equal), project administration (equal), resources (equal), supervision (equal), visualization (supporting), writing – original draft (equal), writing – review and editing (equal).

## Ethics Statement

The animal experiments were approved by the Kumamoto University Animal Experiment Committee and conducted in accordance with the laws and regulations concerning animal experiments, animal care and keeping standards, and basic guidelines.

## Consent

The authors have nothing to report.

## Conflicts of Interest

The authors declare no conflicts of interest.

## Supporting information


**Figure S1:** Structure of components conjugated with GNPs and size properties of nanoparticles.

**Figure S2:** Structure of components conjugated with iGNs and size properties of nanoparticles.

**Figure S3:** Zeta potential of gold nanoparticles.

**Table S1:** RNA‐seq count data.

**Table S2:** DEseq foldchange.

**Table S3:** Downregulated and upregulated differentially expressed genes.

## Data Availability

Raw RNA‐seq data, supporting the findings of the present study, are available from the DNA Data Bank of Japan (DDBJ; accession numbers PRJDB19624). Other data that support the findings of this study are available on request from the corresponding author.
