# Supplementary figures and images for: Peptide Modulation Overrides Glycan Synergy in Gold Nanoparticle‐Based Vaccines for Cancer Immunotherapy
Source: Cancer Med. 2025 Oct 1;14(19):e71286. doi: 10.1002/cam4.71286 (PMC12486328; doi:10.1002/cam4.71286)

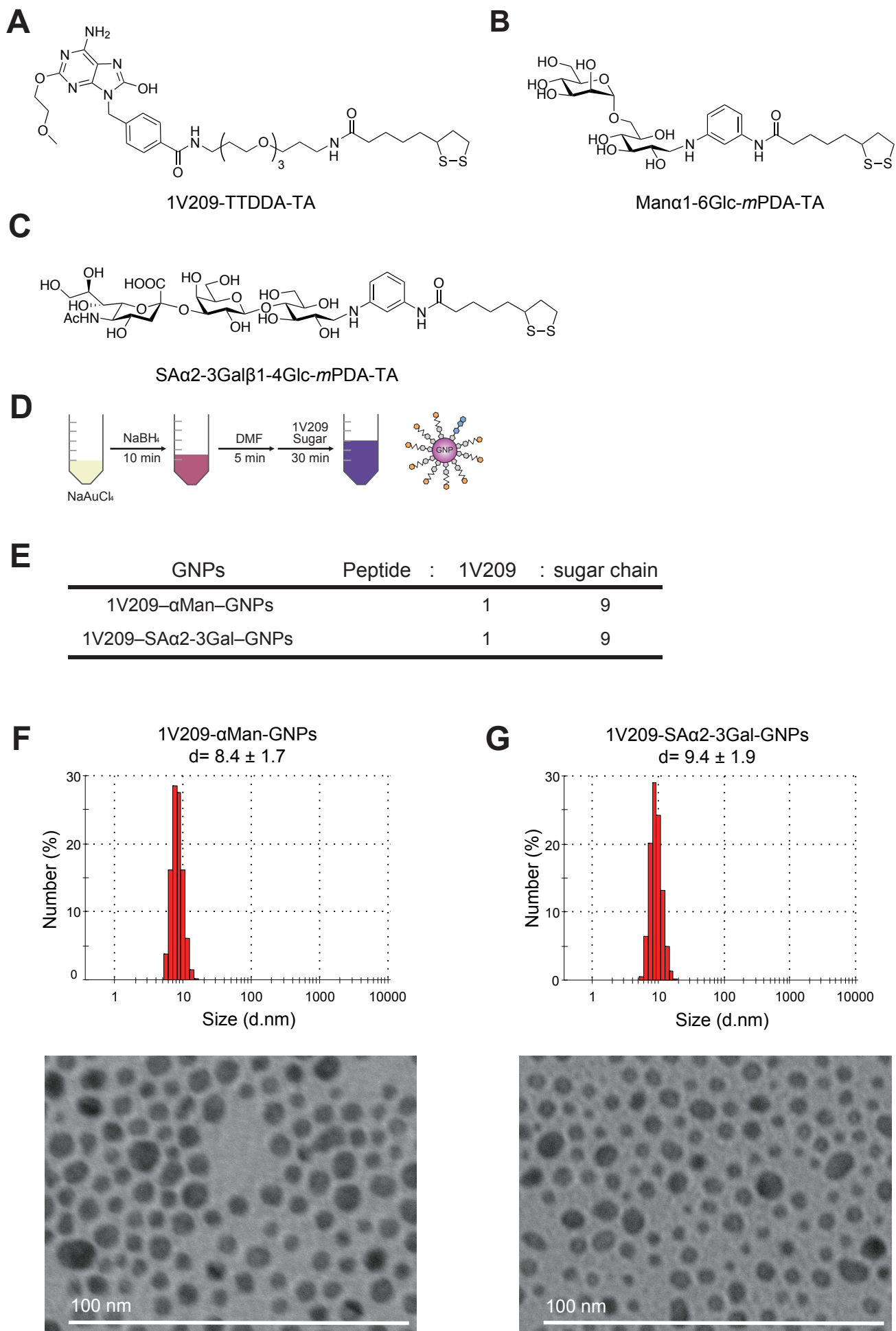

Harada *et al.* Supplementary Figure 1

Supplement: Supplementary file 1 — Figure S1: Structure of components conjugated with GNPs and size properties of nanoparticles. [file CAM4-14-e71286-s002.pdf]

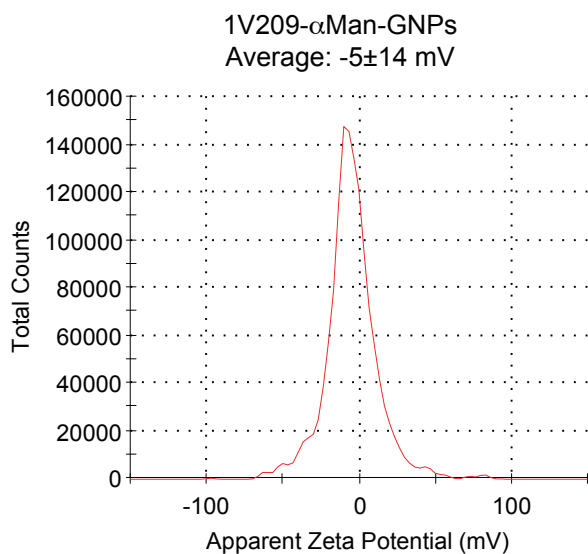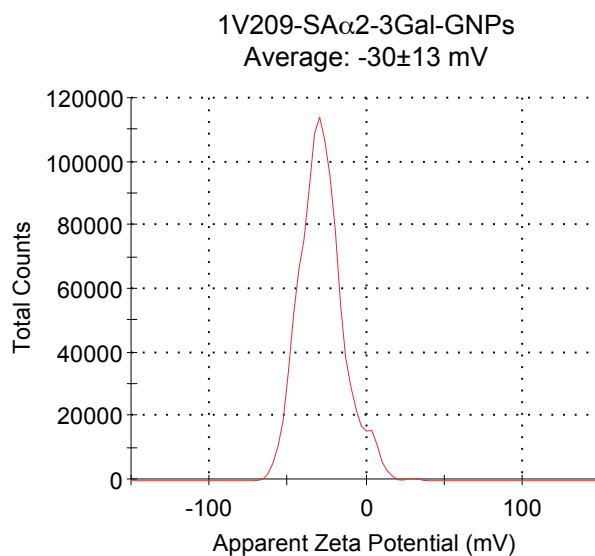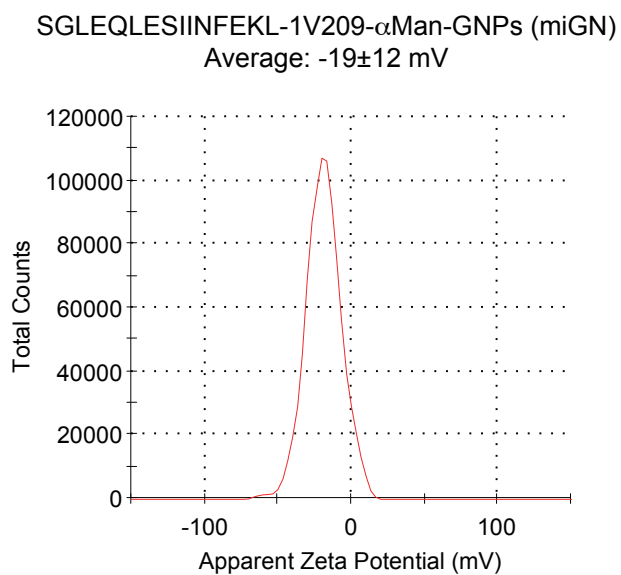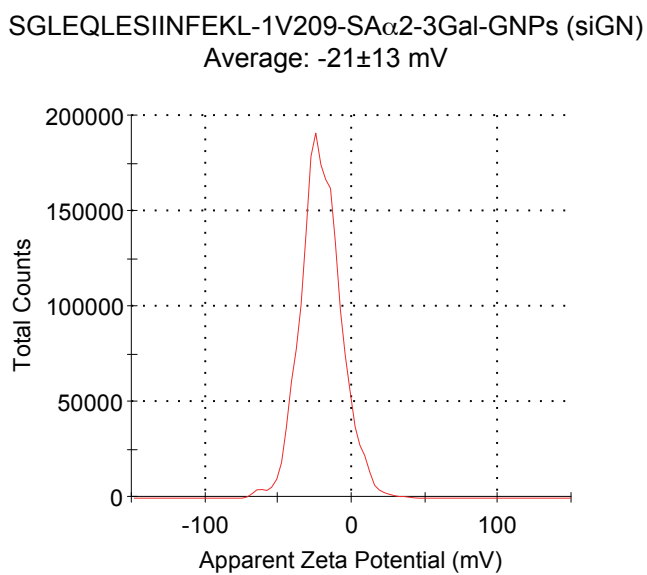

Supplement: Supplementary file 3 — Figure S3: Zeta potential of gold nanoparticles. [file CAM4-14-e71286-s004.pdf]
