# Supplementary material for: Peptide Modulation Overrides Glycan Synergy in Gold Nanoparticle‐Based Vaccines for Cancer Immunotherapy
Source: Cancer Med. 2025 Oct 1;14(19):e71286. doi: 10.1002/cam4.71286 (PMC12486328; doi:10.1002/cam4.71286)
Supplement: Supplementary file 2 — Figure S2: Structure of components conjugated with iGNs and size properties of nanoparticles. [file CAM4-14-e71286-s006.pdf]

A

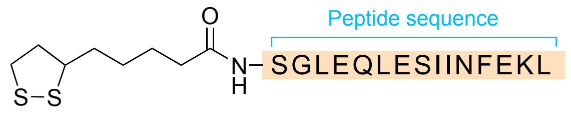

TA-SGLEQLESIINFEKL

B

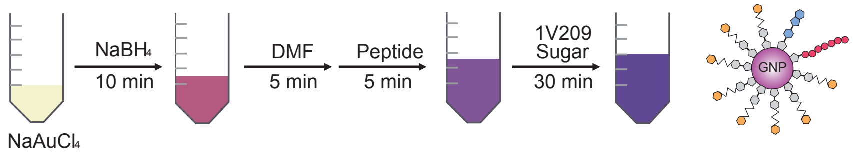

C

GNPs	Peptide :	1V209	: sugar chain
miGN	1	1	8
siGN	1	1	8

D

miGN
SGLEQLESIINFEKL-1V209-αMan-GNPs
d= 11.9 ± 2.7

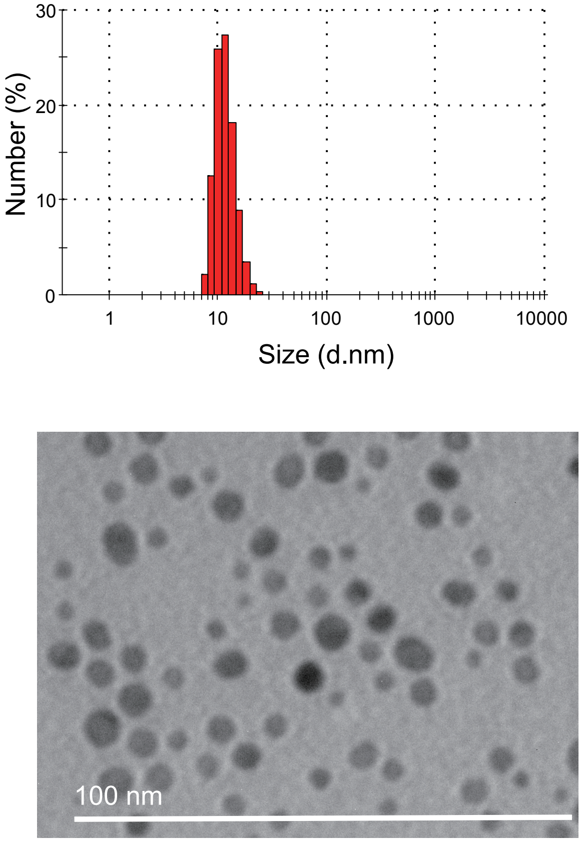

E

siGN
SGLEQLESIINFEKL-1V209-SAα2-3Gal-GNPs
d= 12.3 ± 4.5

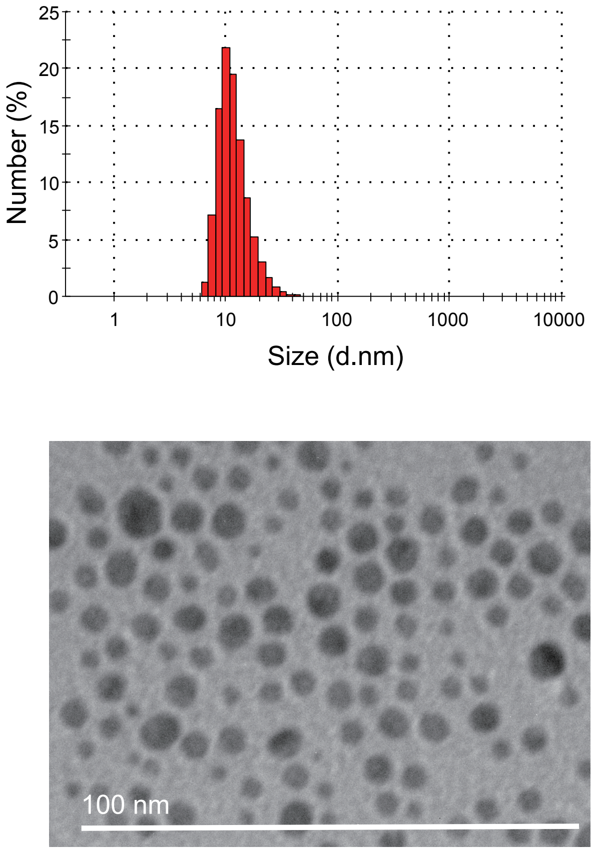

Harada *et al*. Supplementary Figure 2
